# Exploring the Needs and Perspectives of Patients with Obesity to Inform Health Care Practice: A Focus Group Study

**DOI:** 10.3390/jcm15083147

**Published:** 2026-04-20

**Authors:** Gloria Marchesi, Giada Rapelli, Gaia Roselli, Giulia Spina, Michelle Semonella, Gianluca Castelnuovo, Giada Pietrabissa

**Affiliations:** 1 Department of Psychology, Catholic University of Milan, 20123 Milan, Italy; gloria.marchesi@unicatt.it (G.M.); giada.rapelli@unicatt.it (G.R.); gaia.roselli01@icatt.it (G.R.); giulia.spina02@icatt.it (G.S.); michelle.semonella@unicatt.it (M.S.); g.pietrabissa@auxologico.it (G.P.); 2 Clinical Psychology Research Laboratory, IRCCS Istituto Auxologico Italiano, 28824 Piancavallo, Italy

**Keywords:** obesity, focus group, health care, clinical practice, clinical psychology

## Abstract

**Background/Objectives:** This qualitative study investigated the perspectives and lived experiences of individuals with obesity, with a specific focus on psychological needs, beliefs, attitudes, and experiences related to psychological support. The study aimed to identify perceived barriers and facilitators to adherence in weight management and to examine participants’ views on digital psychological interventions designed to promote mental health and well-being. These findings represent the preliminary phase of a broader research project aimed at developing and implementing personalized digital psychological interventions to enhance engagement, treatment effectiveness, and equity of care in obesity management. **Methods:** Five focus groups were conducted with a purposive sample of 35 patients (48.6% female) diagnosed with obesity and enrolled in a four-week multidisciplinary weight-reduction program at the IRCCS Istituto Auxologico Italiano, San Giuseppe Hospital, Piancavallo (VB), Italy. Sessions were audio-recorded, supplemented with field notes, transcribed verbatim, and analyzed using reflexive thematic analysis to identify recurrent patterns of meaning across participants’ narratives. **Results**: Six overarching themes were identified: (1) obesity as an embodied and pervasive experience; (2) the interplay between emotions, weight stigma, and identity construction; (3) family and social relationships as both supportive and ambivalent; (4) personal agency and self-regulation processes in weight management; (5) access to healthcare services and experiences with healthcare professionals; and (6) the perceived role of psychological support within multidisciplinary care. Participants described obesity as a complex, multidimensional condition encompassing physical, emotional, relational, and contextual challenges that directly influence treatment engagement and adherence. **Conclusions**: Psychological support emerged as a central component of comprehensive obesity care. Findings underscore the need for personalized, flexible, and digitally supported psychological interventions to enhance long-term adherence, acceptability, and overall well-being.

## 1. Introduction

Obesity is a multifactorial and potentially life-threatening condition characterized by excess adiposity associated with organ dysfunction and impairments in daily functioning, and is linked to multiple complications, including cardiovascular disease, type 2 diabetes, cancer, and reduced quality of life [1,2]. Its global prevalence is rapidly increasing, with projections estimating a rise from 524 million to 1.13 billion adults between 2010 and 2030, underscoring the urgent need for effective prevention and management strategies [3].

Beyond its biomedical characterization, obesity is increasingly understood as a complex condition influenced by biological, environmental, and behavioral determinants, requiring long-term, multidisciplinary management [4]. A growing body of research highlights the central role of psychological processes in both the development and maintenance of obesity. Individuals living with obesity frequently report depression, anxiety, and chronic stress [5,6], as well as maladaptive eating patterns—such as emotional eating, loss-of-control eating, and other forms of disordered eating. These processes are often embedded within broader lived experiences characterized by weight stigma, internalized bias, and repeated cycles of weight loss and regain, which may undermine self-efficacy and long-term treatment engagement [7,8,9,10]. Qualitative studies have further emphasized how obesity is experienced as an embodied and socially situated condition, shaping identity, interpersonal relationships, and everyday functioning [11,12].

Effective obesity management requires timely, evidence-based, multimodal interventions aimed at reversing the clinical manifestations of the disease and preventing progression to end-organ damage. Recommended treatment strategies include comprehensive lifestyle modification (i.e., dietary changes, increased physical activity, and structured behavioral interventions), pharmacotherapy, and bariatric surgery when clinically indicated [13,14]. Treatment should be individualized, taking into account obesity severity, comorbidities, patient preferences, and prior treatment responses. In parallel, public health policies should ensure equitable access to effective treatments and systematically address weight-based stigma and bias, which remain significant barriers to prevention, healthcare engagement, and long-term treatment adherence [1,2]. Current clinical guidelines, including the updated 2025 guidance from the National Institute for Health and Care Excellence [15], emphasize the central role of tailored behavioral and psychological interventions within comprehensive care pathways [15,16,17]. These interventions aim to support individuals in modifying eating and physical activity behaviors, addressing emotional and cognitive determinants of eating, enhancing self-regulation skills, and promoting sustained adherence and overall well-being, reflecting the recognition of obesity as a chronic, relapsing condition requiring long-term, multidisciplinary management [18].

However, behavioral programs tend to produce clinically meaningful weight loss primarily during the active treatment phase, with long-term follow-up data indicating that many individuals experience partial or substantial weight regain after discontinuing structured support [19].

Furthermore, access to conventional obesity care remains uneven. Engagement in structured and specialized programs is strongly influenced by socioeconomic status, geographic location, and the healthcare system organization. Limited availability of multidisciplinary services, long waiting times, and travel-related constraints disproportionately affect individuals in underserved or rural areas. These barriers underscore the need for more scalable, flexible, and accessible models of care capable of extending support beyond traditional clinical settings [20].

In response to these challenges, digital health solutions are increasingly being explored as cost-effective, accessible, and personalized approaches. These include mobile health applications, web-based platforms, telemedicine, virtual reality tools, and artificial intelligence-driven systems [21,22,23]. Within this landscape, Digital Mental Health Interventions (DMHIs) have emerged as particularly promising, offering flexible and scalable psychological support that can complement conventional multidisciplinary care [24,25,26,27] through tailored content, just-in-time adaptive interventions, and real-time monitoring, thereby reducing logistical and stigma-related barriers associated with face-to-face care [28].

Meta-analytic findings indicate that mobile application-based interventions employing established behaviour change techniques—such as self-monitoring, goal setting, prompts, and personalized feedback—are associated with significant improvements in both weight-related and psychological outcomes. These findings support the integration of digital psychological components into stepped-care obesity models to enhance reach, continuity of care, and long-term engagement [29].

However, despite their potential, the adoption and sustained use of digital interventions remain limited, and their acceptability among individuals living with obesity is not yet fully understood [30].

Importantly, the development and implementation of effective interventions require a comprehensive understanding of patients’ perspectives, experiences, and needs. Qualitative methodologies are particularly appropriate for investigating subjective experiences, especially in areas where phenomena are not yet fully understood, and provide a valuable foundation for the development of patient-centered interventions [31]. While a growing body of research has explored these aspects in different contexts [32,33,34], highlighting the importance of tailoring digital support to specific needs and contexts—including preferences for personalized content, self-monitoring tools, and integration with clinical care—evidence focusing on the Italian population remains limited, particularly with regard to individuals’ perspectives on psychological support and digitally delivered interventions. Addressing this gap is essential to inform the development of interventions that are responsive to the specific socio-cultural and healthcare context in which they are implemented.

Addressing this gap, the present study employs a qualitative focus group design to explore the perspectives of individuals living with obesity regarding psychological support in weight management. Specifically, the study aims to (1) explore participants’ experiences, beliefs, and attitudes related to obesity management and its psychological impact, and (2) investigate patients’ perspectives on psychological support for weight management, with particular emphasis on the acceptability and perceived value of digitally delivered interventions.

Through an in-depth examination of participants’ expectations, perceived needs, and attitudes toward psychological and digitally delivered support, this study aims to generate practice-oriented insights to inform the development, structure, and delivery of a digital psychological self-help intervention for individuals living with obesity. Grounded in a patient-centered framework, the project is designed to ensure that the intervention is both theoretically robust and aligned with real-world clinical practice.

## 2. Materials and Methods

### 2.1. Design

A qualitative research design using focus groups was adopted to explore participants’ experiences, beliefs, and attitudes related to obesity and its psychological impact, as well as perceived barriers and facilitators to psychological support, with particular attention to attitudes toward digitally delivered interventions. The qualitative approach was selected because it is widely recognized as a valuable method for informing the development of patient-centered interventions in the context of emerging or underexplored phenomena. In particular, focus groups were selected to collect qualitative data in a group setting, facilitating discussion and reflection on participants’ experiences and perceptions [35,36].

The study was approved by the Ethics Committee of the IRCCS Istituto Auxologico Italiano, Milan, Italy (ID: 03C101_2011), and was in accordance with the Helsinki Declaration of 1975, as revised in 2008. The Consolidated Checklist for Reporting Qualitative Research (COREQ) was used to ensure methodological rigor and transparency [37] (see Supplementary File S1). The purpose of the study was explained to each subject, and written informed consent was obtained from all participants before the study began.

### 2.2. Participants

A purposive sample of 35 patients (48.6% female; age range 21–66 years; M = 50.49, SD = 12.24) was recruited in person from the IRCCS Istituto Auxologico Italiano, San Giuseppe Hospital, Piancavallo (VB), Italy. Participants were enrolled in a multidisciplinary inpatient nutritional rehabilitation program for obesity lasting approximately 28 days. Inclusion criteria were: (1) a BMI ≥ 30 kg/m^2^, (2) 18 years of age or older, and (3) Italian language proficiency. Participants with current psychiatric diagnoses assessed through routine clinical psychiatric evaluation and diagnostic interviews conducted by the hospital team at inclusion in the clinic were excluded. Individuals participated in five focus groups, each with 6–8 participants. No dropouts occurred (see Table 1 for demographic and clinical information).

### 2.3. Procedure

Data were collected from November 2025 to December 2025 until data saturation was reached, defined as the point at which no new themes or codes emerged across the focus group discussions [38].

Face-to-face focus group sessions were moderated by a research psychologist with expertise in focus group facilitation (G.M.), who had no prior contact with participants, and were accompanied by two observers (G.R. and G.S.). The observers did not actively participate in the discussion but independently recorded field notes on group dynamics, non-verbal communication, and the overall emotional climate. Following each session, the moderator and observers conducted a debriefing to discuss emerging impressions and to integrate observational data into the analytic process.

Focus groups were conducted in a designated hospital room outside standard working hours, audio-recorded, and lasted 55–90 min. Participants were informed that participation was voluntary, that they could withdraw at any time, and that results would be used for scientific publication. No compensation or reward was provided.

A semi-structured interview guide (see Supplementary File S2) was used to explore experiences, beliefs, knowledge, and perceptions regarding psychological support in weight management. A storytelling approach with probing questions (e.g., “Tell me more about that experience.”; “How did that make you feel?”) was employed to elicit in-depth discussion and facilitate a dialogic interaction [39].

### 2.4. Data Analysis and Rigor

Focus groups represent a particularly rich source of experiential data [40], as they (1) enable the collection of multiple perspectives to be captured within a single session, allowing the inclusion of a larger sample with fewer data collection events, (2) facilitate deeper experiential reflection compared with one-on-one interviews, and (3) are cost-effective within hospital settings.

All audio recordings were transcribed verbatim and independently coded by two researchers (G.R. and G.S.), without the use of qualitative analysis software. Field notes were also collected to capture contextual information and overall impressions during each session. Transcriptions were analyzed qualitatively using thematic analysis [41], adopting a data-driven approach, which is considered most effective for exploring subjective experiences [42].

The analytical procedure began with a line-by-line reading of each transcript to initial codes and a descriptive summary of relevant content. This iterative process involved repeated reading of the transcript—first within each focus group, then across groups—to allow for the emergence of conceptually meaningful themes. Initial codes were then clustered into higher-order themes.

Coding was conducted independently by two researchers (G.R. and G.S.), and consensus on all themes was achieved through discussion. Any remaining disagreements were resolved by a third independent researcher (G.M.).

In total, 113 codes were generated and subsequently organized into six overarching themes. The analysis aimed to faithfully reflect participants’ narratives, ensuring that all themes were strongly grounded in the data. Participants were not involved in the analytical process, and no member-checking procedure was conducted to validate the findings.

## 3. Results

### 3.1. Obesity as an Embodied and Totalizing Experience

Participants reported that living with obesity involves multiple physical and functional challenges that substantially affect daily activities. These difficulties contribute to the perception of obesity as a pervasive and totalizing experience, compromising overall quality of life, shaping daily routines, limiting participation in activities, and reinforcing the centrality of the body in one’s identity. Reported challenges included reduced mobility and practical difficulties in everyday tasks.

“… *for me, walking has become difficult*.” (P1, P6)

“*Every morning when I get up, putting on or tying my shoes is difficult*.” (P22, P30)

Participants also described physical symptoms, such as shortness of breath, as well as pain and limitations associated with comorbid conditions or medical procedures.

“*I often fainted while walking, due to lack of breath.*” (P1)

“*I had two surgeries, and I have difficulties due to pain.*” (P17, P21)

Environmental and practical barriers were also emphasized. Some participants highlighted the inadequacy of physical and social environments for individuals living with obesity, as well as challenges in finding appropriate clothing.

“*… you are on vacation and cannot fit into the shower.*” (P5)

“*You have to deal with a world that is no longer suited to us. Small chairs, uncomfortable seats on airplanes.*” (P7)

Behavioral and lifestyle factors were described as contributing to these functional challenges. In particular, some participants reported that pharmacological treatments for comorbid conditions (e.g., endocrine disorders) increased appetite and facilitated weight regain, despite previous weight-loss efforts.

“*If you have another illness that forces you to take strong medications, you realize that all the effort you made to lose those 10 kg is lost—in just a couple of months, I gained them back.*” (P1)

### 3.2. From Emotions and Stigma to Identity

Participants described a range of emotional experiences, including emotional overload and feelings of guilt. These emotions appeared to shape individuals’ sense of identity and self-perception, contributing to self-doubt and heightened self-criticism, while also influencing motivation, decision-making, and engagement in weight management behaviors.

“*At home, you also end up acting as a cushion for others, absorbing harsh remarks and observations, while at the same time having a family to take care of. At a certain point, I just collapse.*” (P1)

Perceived traumatic experiences (e.g., bereavement or serious illness of a loved one) emerged as significant factors contributing to emotional eating behaviors and weight-gain trajectories. These findings highlight a maladaptive cycle in which emotional eating may function as a primitive coping strategy, particularly during vulnerable life transitions and stressful events. As a consequence, individuals’ sense of efficacy in managing difficulties in a more adaptive way may be undermined. Over time, this may contribute to the development of an identity centered on a perceived inability to control weight gain, accompanied by broader self-attribution of failure.

“*In October, my 32-year-old nephew died of melanoma … experiences like that inevitably make you turn to food for comfort.*” (P4)

Body-related perceptions and internalized stigma, such as shame, self-criticism, and feelings of inadequacy, were reported to significantly affect participants’ identity. These experiences led some individuals to avoid social situations or activities—including interpersonal interactions, work-related contexts, and intimate relationships—due to concerns about others’ judgments or anticipated physical discomfort.

“*Sometimes I avoided doing certain things just because of my body. I would think, ‘What might they think, that I’m overweight?*’” (P26)

“*It all started as a kind of workplace bullying—you know, someone makes a joke, and then the other person reacts, and it escalates …*” (P24)

“*There was discomfort in being intimate with my wife.*” (P20)

### 3.3. Family and Social Relationships: Supportive Yet Challenging

Participants highlighted the central role of family, social, and cultural contexts in shaping eating behaviors and the weight management process. Family relationships emerged as both a facilitating and constraining factor in lifestyle changes.

Supportive family dynamics were described as particularly beneficial when significant others adopted healthier habits alongside the participant.

“*My family is very supportive.*” (P30)

“*Now my husband has changed as well … and he feels better too.*” (P1)

At the same time, participants noted that family composition and responsibilities could limit the feasibility of dietary changes.

“*With teenage children … You can’t expect them to eat the same way you do.*” (P2)

Social relationships were also reported to play a significant role, particularly in relation to food-centered socialization. Some participants reported distancing themselves from certain friendships to protect their health and maintain behavioral changes.

“*That’s why I let that friendship go, exactly for that reason. We used to meet at someone’s house, and there was always food … so I ended up keeping only four friends.*” (P1)

More broadly, participants emphasized the cultural significance of food in social life, noting that, in the Italian context, social interactions are often closely tied to eating and drinking. This cultural norm may pose additional challenges to adherence to dietary changes.

“*Socializing, in Italy, usually happens around something to eat or drink.*” (P2)

### 3.4. Personal Agency and Self-Regulation in Weight Management

Motivation was described as a key factor in the weight management process and in sustaining behavioral changes. When expectations were not met, or progress was perceived as insufficient, participants reported experiencing feelings of failure and engaging in increased self-criticism, which appeared to undermine self-efficacy. This dynamic, in turn, may negatively affect long-term engagement in treatment.

“*It depends on me, because the doctors can give all the advice in the world, but if I get home and stop by restaurant X, it’s pointless!*” (P29)

Challenges related to eating behaviors included emotional eating, cravings, and the hedonic value associated with food.

“*I wanted chocolate out of boredom …*” (P16)

“*I enjoy eating, and I like the flavors.*” (P29)

External factors, such as organizational and work-related constraints, were described as further complicating dietary adherence and daily routines.

“*At work, I don’t have a fixed schedule; sometimes I eat mid-morning, sometimes mid-afternoon, and then I risk eating late in the evening—it throws everything off.*” (P17)

Setbacks and fluctuations in motivation were frequently reported as a natural part of the change process.

“*Maintaining the diet is difficult.*” (P3)

“*Laziness is the biggest difficulty.*” (P1, P2)

To cope with these challenges, participants described implementing self-management strategies, including preparing and portioning food in advance.

“*Having everything ready, the little bags and measured pasta portions … I froze them, and when I was in a hurry, I would just take one.*” (P18)

Mindful eating was also mentioned as a strategy to support weight management.

“*I practiced mindful eating to manage my weight.*” (P31)

Finally, participants highlighted the cumulative effect of multiple dieting attempts over time, which often contributed to frustration and made sustained change more difficult.

“*I have always experienced diet attempts as a major frustration, because since I was a teenager, I have been trying to control my weight through doctors and various treatments.*” (P31)

“*It’s like a vicious circle, and it just keeps getting worse.*” (P14)

### 3.5. Access to Healthcare Services and Experiences with Professionals in Weight Management

Participants reported that access to healthcare services for weight management was often limited by logistical, practical, and economic barriers.

Some participants described difficulties in accessing services due to geographic distance or mobility constraints. In this context, continuity of care through accessible follow-up after hospitalization was perceived as particularly important for monitoring progress and providing timely support, thereby potentially reducing the risk of disengagement or relapse.

“*I looked for them, but they were all far away … they suspended my driving license … I can’t reach anyone anymore.*” (P1)

The perceived quality of communication with healthcare professionals also emerged as a crucial factor in weight management. Participants emphasized the importance of empathy, attentiveness, and a multidisciplinary approach. In contrast, inadequate communication was described as a barrier to treatment adherence and open dialogue, as patients may feel reluctant to seek clarification or discuss difficulties, particularly when interactions are perceived as judgmental or stigmatizing.

“*There should be more attention to people … from all the doctors, everyone … they don’t have tact.*” (P12)

“*Having a team behind you is useful.*” (P13)

### 3.6. Psychological Support in Weight Management

#### 3.6.1. Perceived Importance of Psychological Support in Weight Management

Participants highlighted the crucial role of psychological support in the management of obesity.

Many recognized that addressing psychological aspects is as important, or even more important, than physical interventions. This perspective reflects a holistic understanding of obesity and underscores the need for integrated, multidisciplinary care to achieve effective outcomes.

“*In my opinion, a psychologist can help. I have experienced it personally.*” (P2)

“*I think the psychological aspect is even more important than the physical one, because the first step of any dietary change lies in psychological and behavioral processes.*” (P9)

“*Obesity is a psychological matter.*” (P14)

At the same time, participants reported several barriers to accessing appropriate psychological support. Geographic limitations, environmental conditions, lack of information, and financial constraints were identified as key obstacles.

“*I live in a small town: it’s not certain that I can find the psychologist most suitable for me, so maybe I have to travel to the nearby city; weather conditions, my knee, and other factors make it difficult.*” (P2)

“*Perhaps they could offer people with obesity some advantages, such as reduced fees for those suffering from these conditions.*” (P9)

In addition, some participants expressed concerns about becoming dependent on psychological support or reported difficulties in establishing a stable and continuous therapeutic relationship. These concerns may reflect underlying bias or misconceptions about psychotherapy, potentially affecting engagement.

“*If you go to the psychologist, it becomes a vortex—you can’t manage without it.*” (P23)

“*For example, I was entitled to a psychologist for my cancer: only a few sessions, and then … I looked at it and said no, I’ll avoid it, because if you want to continue, you have to redo the other queue, maybe it’s no longer the same psychologist …*” (P2)

Participants emphasized that continuity of care and the quality of the therapeutic alliance are essential for psychological support to be effective and meaningful in weight management.

#### 3.6.2. Preferences and Expectations for Psychological Support Services

Participants reported clear expectations regarding psychological support for weight management. Several emphasized the importance of working with a psychologist specialized in obesity and valued a dialogical, interactive approach during sessions.

“*The psychologist should be specialized in managing obesity.*” (P1, P13)

“*I want the psychologist to speak as well.*” (P19)

Participants also highlighted the need for psychological support addressing eating behaviors and difficulties in self-regulation.

“*For people with eating disorders, even a single word can make a difference and completely change things. Psychological support is essential, because I cannot know how to get out of an illness on my own without professional help.*” (P13)

Emotional processes related to eating were described as central targets of intervention.

“*The psychologist should help me bring out my fears.*” (P28)

Several participants emphasized the importance of addressing body image-related difficulties, including distorted body perception and dissatisfaction.

“*We tend to see ourselves in the mirror differently from how we actually are, and this creates a distorted perception of our body. This is important to address with psychological support.*” (P12)

Psychological support was also perceived as a resource for enhancing self-awareness and sustaining motivation over time.

“*The psychologist is someone who gives me strength.*” (P1)

Individualized support was strongly preferred over group-based formats, along with flexibility in the duration and structure of interventions.

“*The psychological pathway should be individual, lifelong.*” (P2, P5, P9, P12)

“*There shouldn’t be a fixed duration: one session may be enough for me, three for you, two for someone else …*” (P1)

Views on group interventions were mixed. Some participants valued the opportunity for sharing experiences, whereas others perceived group settings as potentially uncomfortable or emotionally demanding.

“*I see group sessions more for comparison: my experience, theirs, maybe it helps …*” (P31)

“*Group sessions can be as useful as harmful, because not everyone is used to listening to other people’s stories, and sometimes it can create discomfort.*” (P13)

Overall, participants emphasized that psychological support should be personalized and adaptable to individual emotional and practical needs.

#### 3.6.3. Engagement with and Preferences for Digital Psychological Support Tools

Participants reported mixed attitudes towards digital psychological support. On the one hand, some participants expressed openness to online interventions, describing them as useful in overcoming logistical and environmental barriers.

“*Being in person requires driving, parking, and climbing stairs … digital support would be useful.*” (P2)

“*I would try a digital intervention.*” (P2, P3, P12)

On the other hand, several participants emphasized a preference for face-to-face interaction, highlighting both relational factors and difficulties with technology use.

“*In my opinion, in-person is better … You see me, you help me. I like face-to-face interaction.*” (P1)

“*Online, absolutely not, because I can’t handle technology.*” (P22)

Hybrid formats were suggested as a potential solution to balance accessibility and relational quality.

“*One could alternate between online and in-person sessions.*” (P1)

“*It’s not always possible to find a face-to-face psychologist who suits me best, so I might have to travel to a nearby city. Weather conditions, like snow or rain, can make it harder, and sometimes I’m just feeling lazy. There are also other factors, like the cold, the heat, or my knee …*” (P2)

Digital tools were also perceived as valuable for supporting self-management. Participants noted that exercises, homework, and psychoeducational materials could facilitate reflection and behavioral change.

“*Having tasks, exercises, studying them to understand where you make mistakes could also be useful …*” (P3)

The possibility of receiving timely support during crises was considered a key advantage of digital interventions.

“*But now that it’s possible to do it via video call, it wouldn’t be bad. Maybe I have a crisis today, and she is available in twenty days: we’ll never be in the same moment. So, having support on the other side—that helps you understand that the ‘emergency’ (so to speak) is there—wouldn’t be bad. Because you get it at the moment you need it.*” (P1)

Attitudes toward digital support appeared to vary by age, with younger participants generally more open to online interventions and older individuals more likely to prefer in-person formats. Overall, participants emphasized that psychological support—whether in-person, digital, or hybrid—should be flexible, personalized, and responsive to both emotional and practical needs.

The emerging themes and related illustrative quotes are reported in Table 2.

## 4. Discussion

To our knowledge, this is the first qualitative study conducted in Italy exploring experiences, beliefs, and attitudes toward psychological support among individuals with obesity, with a specific focus on digitally delivered interventions. While previous qualitative studies have primarily focused on behavioural outcomes such as weight loss and lifestyle change [43,44], the present study extends this literature by incorporating patients’ perspectives on digital support, thereby informing the development of patient-centered interventions.

Overall, the findings portray obesity as a pervasive experience that affects multiple domains of everyday life, shaping daily functioning, emotional experiences, and social relationships. Participants consistently described obesity as an embodied and intrusive condition, rather than merely a clinical state [11,12]. The present findings further highlight how these embodied experiences are intertwined with environmental and contextual constraints, such as inadequately designed physical spaces and limited accessibility of services. These factors reinforce the perception of obesity as a condition that permeates both the body and the surrounding environment, ultimately influencing individuals’ sense of agency and autonomy. This multidimensional impact aligns with contemporary conceptualizations of obesity as a chronic and complex condition involving functional impairment and psychosocial burden [2,45]. Indeed, contextual barriers may exacerbate obesity-related impairment and further reduce perceived quality of life [4].

Emotional experiences emerged as central and pervasive in participants’ narratives. Feelings of emotional overload, guilt, and stigma were frequently reported and appeared to threaten individuals’ sense of self. Consistent with prior qualitative research [46], stigma was described across multiple contexts, including interpersonal relationships and healthcare interactions, with significant consequences for self-perception and help-seeking behaviors. Participants also reported internalized stigma, which negatively influenced social participation and intimate relationships. Emotional suffering appeared both as a consequence of obesity and as a constitutive component of participants’ self-understanding in line with previous studies [47,48].

The present findings extend this literature by illustrating how such experiences may become integrated into identity construction, fostering self-critical narratives and reinforcing disengagement from care.

The role of social and relational contexts was similarly complex. Family and social relationships were described as both supportive and constraining, reflecting an ongoing tension between the need for belonging and the demands of behavioral change. Consistent with previous evidence, partner involvement has been shown to enhance treatment outcomes through social support, modeling of healthy behaviors, and management of high-risk situations [49]. Moreover, supportive environments facilitated adherence to lifestyle modifications, whereas social norms and food-centered interactions posed challenges [50].

These findings underscore the importance of considering the broader socio-cultural context in designing weight management interventions, particularly in Italy, where food plays a central role in social life.

Participants also reported substantial challenges related to self-regulation and long-term behavior change. Repeated unsuccessful weight management attempts contributed to frustration, reduced self-efficacy, and difficulties in maintaining motivation. Consistent with previous studies [51,52,53], eating behaviors were characterized by emotional eating, cravings, and the hedonic value of food, alongside organizational constraints related to work schedules and daily routines. These patterns may reflect disruptions in self-regulation processes, whereby repeated cycles of weight loss and regain undermine perceived control and long-term engagement. The tension between desire for control and perceived helplessness, often interpreted as personal failure, may further erode motivation and contribute to negative self-definition. At the same time, participants reported adopting adaptive strategies, such as meal preparation and structured routines, highlighting the coexistence of barriers and resources within the change process.

Barriers to accessing healthcare services were widely reported and included logistical, geographic, and economic constraints, as well as dissatisfaction with patient–provider communication. Participants emphasized the importance of empathy, continuity of care, and multidisciplinary approaches, in line with current clinical recommendations for integrated and obesity care [15,54]. Notably, these barriers often interact with individual and contextual factors, further complicating sustained engagement with treatment.

Within this framework, psychological support was consistently perceived as a key component of obesity care. Participants described the psychologist not only as a provider of behavioral strategies but also as a relational figure offering emotional containment, guidance, and validation. Individual psychological support was generally preferred over group formats, highlighting the importance of personalized approaches in promoting engagement and effectiveness [29]. These findings reinforce the need for psychological interventions that extend beyond behavior-change techniques to address emotional, identity-related, and relational processes, in line with evidence on tailored psychological approaches [55,56,57].

At the same time, barriers to psychological support were identified, including concerns about dependency on therapy, logistical and financial constraints, and discontinuity of care. These findings highlight the need for flexible and sustainable psychological support [20].

In this context, digitally delivered psychological interventions were perceived as both promising and challenging. Participants valued digital tools for their potential to overcome logistical barriers, provide timely support during critical moments, and enhance flexibility.

These findings are consistent with prior qualitative research showing that remote support can foster accountability, motivation, and engagement in weight management [43,44]. Participants in the present study emphasized similar benefits, valuing personalized, flexible digital support. However, concerns were raised regarding technological literacy, reduced interpersonal connection, and a preference for face-to-face interaction, particularly among older participants. These findings suggest that the acceptability of digital interventions may depend on individual characteristics and contextual factors and support the development of hybrid models combining in-person and digital components. Importantly, digital tools were viewed as complementary rather than substitutive, consistent with evidence positioning digital mental health interventions as adjuncts to traditional care [30].

Participants also highlighted the utility of digital tools for self-management, including homework, exercises, and psychoeducational materials that could foster reflection, awareness, and continuity between sessions [28]. Overall, these findings suggest that digital psychological interventions may enhance accessibility and continuity of care when designed to complement relational aspects of treatment [21].

Taken together, this study provides context-specific insights into how individuals living with obesity perceive psychological support and its potential digital delivery. In particular, the findings highlight the need to move beyond standardized approaches and to develop flexible, personalized interventions responsive to both psychological and practical needs. The integration of digital components into obesity care pathways should therefore consider not only scalability and efficiency, but also patients’ preferences, capabilities, and relational expectations.

From a clinical perspective, interventions should prioritize continuity of care, personalization, and the integration of psychological support within multidisciplinary frameworks. Digital solutions may play a valuable role in extending access and supporting self-management, particularly when designed to complement face-to-face interactions. Features such as tailored content, real-time support, and opportunities for relational engagement may enhance acceptability and effectiveness.

### Strengths and Limitations of the Study

This study has several limitations that should be acknowledged. First, the use of a purposive sample recruited from a single inpatient multidisciplinary obesity program may limit the generalizability and transferability of the findings. However, the relative homogeneity of the sample may have enhanced the depth and coherence of shared experiences, strengthening the exploratory value of the results. The inclusion of a gender-balanced sample with a wide age range allowed for the capture of diverse perspectives on living with obesity. Although the study explored attitudes toward digital interventions for psychological support, their effectiveness was not assessed and requires further investigation. As with all qualitative research, findings are context-dependent and should be interpreted as exploratory. Future studies should adopt mixed-methods designs to further investigate patients’ perspectives on digital psychological interventions and examine how such approaches can be effectively integrated into existing care pathways. Greater attention should also be given to diverse populations and settings to better understand the influence of socio-cultural and structural factors on engagement with both traditional and digital forms of support.

## 5. Conclusions

This study highlights that living with obesity is a multifaceted experience shaped by physical, emotional, relational, and contextual factors that influence engagement in weight management and psychological support. Participants emphasized the central role of psychological support while also identifying significant barriers related to access and continuity of care. Although face-to-face support was often preferred, participants expressed openness to flexible and alternative modalities, particularly when these enhance accessibility and responsiveness to psychological needs. Overall, these findings underscore the importance of developing patient-informed, scalable psychological interventions to improve acceptability, engagement, and continuity of care in obesity management.

## Figures and Tables

**Table 1:** Demographic and clinical information about the sample.

	Total Sample (*n* = 35)	
	*n*	%
Gender		
Male	17	48.6
Female	18	51.4
	M (range)	SD
Age (years)	50.49 (21–66)	12.24
BMI (Kg/m^2^)	44.81 (31.11–66.12)	8.45

**Table 2:** Emerging themes and related illustrative quotes.

Themes	Quotations	Respondents (*n*)
1. Obesity as an embodied and totalizing experience	“I have trouble tying my shoes or putting them on.” (P22)	23
2. From emotions and stigma to identity	“I realize that many times they look at me a bit with that face, like saying ‘poor thing, I wonder how they manage’ … And inside me, even though I feel strong, it hurts because it touches my pride; it’s like anger.” (P14)	16
3. Family and social relationships: supportive yet challenging	“My partner helped me a lot … We got rid of anything at home that could be tempting, and since she’s a cook, she started preparing only healthy meals for both of us.” (P7)	14
4. Personal agency and self-regulation in weight management	“If you have a setback, you shouldn’t demonize yourself; you shouldn’t be hard on yourself.” (P12)	24
5. Access to healthcare services and experiences with professionals in weight management	“It means a lot to have a professional who encourages you rather than criticizing you for not losing 1 kg. But if they start saying ‘oh, so you slipped up,’ and maybe you didn’t, it just confuses you a bit—at least it does me, I can’t speak for others.” (P1)	16
6. Psychological support in weight management	“The psychologist is a professional who knows exactly what to do and which words to use at the right moment, because with someone who has an eating disorder, just one word can change everything.” (P13)	29

## Data Availability

The data presented in this study are available on reasonable request from the corresponding author.

## Supplementary Materials

The following supporting information can be downloaded at: https://www.mdpi.com/article/10.3390/jcm15083147/s1, S1: COREQ Checklist, S2: semi-structured interview
