# Supplementary material for: Exploring the Needs and Perspectives of Patients with Obesity to Inform Health Care Practice: A Focus Group Study
Source: J Clin Med. 2026 Apr 20;15(8):3147. doi: 10.3390/jcm15083147 (PMC13116844; doi:10.3390/jcm15083147)
Supplement: Supplementary file 1 [file jcm-15-03147-s001.zip › Supplementary file S2 Semistructured interview.pdf]

SEMI-STRUCTURED INTERVIEW

Domain	Questions	Rationale
Daily life	• How would you describe your daily life while trying to manage your weight?	An ice-breaking question to explore lived experience and everyday functioning, consistent with qualitative approaches focusing on subjective meaning-making.
	• Could you share a particularly difficult moment you have experienced?	To elicit emotionally salient experiences and identify critical incidents shaping behavior and engagement
Treatment experiences	• How have you experienced weight management programs (medical, nutritional, psychological)?	To understand prior treatment experiences and contextualize attitudes toward care
	• What aspects were most helpful, and what were less useful or more challenging?	To explore perceived facilitators and barriers without assuming causal insight
	• What improvements or additions would you suggest for these programs?	To explore barriers and contextual constraints
Psychological support	• How was psychological support helpful (if received)? What could be improved?	To explore the perceived value and meaning of psychological support and identify expectations and representations
	• What benefits or potential risks do you perceive in receiving psychological support for weight management?	
	• If you have never received psychological support, how do you imagine it? What would you expect or hope for?	
	• What emotions, experiences, or situations motivated you to seek psychological support?	To identify drivers and barriers to engagement
	• Who or what prompted you to begin treatment?	
	• What difficulties did you encounter in seeking or accessing psychological support?	To explore structural and personal barriers
	• Who or what discouraged you from seeking psychological support?	
Future expectations	• In your opinion, what characteristics would make psychological support useful and effective in weight management? (e.g., content, duration, frequency, language, involvement of family members)	To explore desires and capture perceived satisfaction with the received psychological support and any perceived gaps, to design future interventions
Delivery preferences (including digital)	• How would you prefer to receive psychological support? (e.g., online, in person, individually, in a group, via app or chat, short or more in-depth sessions)	To explore the acceptability of delivery modalities
Digital interventions	• What are the pros and cons of digital psychological support?	The more open question to explore the acceptability of digital intervention and their expectations regarding new delivery formats to design with participants future interventions
Additional information	• Is there any other information, experience, or anecdote that you think is important to share?	To allow the emergence of unanticipated themes
